# Supplementary material for: Physical Activity after Colorectal Cancer Diagnosis and Mortality in a Nationwide Retrospective Cohort Study
Source: Cancers (Basel). 2021 Sep 25;13(19):4804. doi: 10.3390/cancers13194804 (PMC8508146; doi:10.3390/cancers13194804)
Supplement: Supplementary file 1 [file cancers-13-04804-s001.zip › cancers-1348489-supplementary.pdf]

Table S1. Weekly Frequency of Moderate-to-Vigorous Post-diagnosis Physical Activity and All-Cause/Colorectal Cancer/Cardiovascular Mortality in Colon and Rectal Cancer Patients.

Treatment	PA	*n*	Person-years	All-Cause			Colorectal Cancer			Cardiovascular		
				Events	HR	(95% CI)	Events	HR	(95% CI)	Events	HR	(95% CI)
**Colon cancer**												
		27,152	66,479.30	1674			1156			51		
Total	0–2	16,411	39,649.36	1127	1 (Ref)		757	1 (Ref)		40	1 (Ref)	
	3+	10,741	26,829.94	547	0.84	(0.75, 0.93)	399	0.86	(0.76, 0.98)	11	0.67	(0.33, 1.34)
		20,235	51,621.64	754			380			44		
Surgery only	0–2	12,353	31,043.92	539	1 (Ref)		269	1 (Ref)		35	1 (Ref)	
	3+	7882	20,577.71	215	0.84	(0.71, 0.99)	111	0.86	(0.68, 1.09)	9	0.65	(0.31, 1.40)
		6726	14,507.77	842			704			7		
Surgery with chemotherapy and/or radiotherapy	0–2	3935	8392.96	536	1 (Ref)		439	1 (Ref)		5	1 (Ref)	
	3+	2791	6114.81	306	0.82	(0.71, 0.95)	265	0.85	(0.72, 1.00)	2	0.70	(0.13, 3.86)
		191	349.90	78			72			0		
Chemotherapy with or without radiotherapy	0–2	123	212.48	52	1 (Ref)		49	1 (Ref)		0		
	3+	68	137.42	26	0.93	(0.56, 1.54)	23	0.84	(0.50, 1.44)	0		
**Rectal cancer**												
		16,464	40,499.64	973			687			27		
Total	0–2	10,071	24,308.42	682	1 (Ref)		472	1 (Ref)		17	1 (Ref)	
	3+	6393	16,191.23	291	0.75	(0.65, 0.87)	215	0.75	(0.64, 0.89)	10	1.24	(0.54, 2.84)
		9998	26,027.91	320			149			19		
Surgery only	0–2	6141	15,651.52	242	1 (Ref)		113	1 (Ref)		13	1 (Ref)	
	3 +	3857	10,376.39	78	0.72	(0.55, 0.94)	36	0.74	(0.50, 1.10)	6	0.90	(0.32, 2.53)
		5972	13,677.37	552			447			8		
Surgery with chemotherapy and/or radiotherapy	0–2	3630	8211.80	377	1 (Ref)		302	1 (Ref)		4	1 (Ref)	
	3+	2342	5465.57	175	0.76	(0.63, 0.91)	145	0.75	(0.61, 0.92)	4	2.57	(0.59, 11.19)
		494	794.36	101			91			0		
Chemotherapy with or without radiotherapy	0–2	300	445.10	63	1 (Ref)		57	1 (Ref)		0		
	3+	194	349.26	38	0.87	(0.55, 1.37)	34	0.83	(0.51, 1.34)	0		

Analysis with frequency of moderate-to-vigorous physical activity. Fully Adjusted for age at diagnosis, sex, BMI, smoking, alcohol consumption, insurance premium, Charlson comorbidity index, pre-diagnosis physical activity. For the total patients, treatment options were also adjusted. PA, physical activity; *n*, number of patients; HR, hazard ratio; 95% CI, 95% confidence interval; Ref, reference.

Table S2. Pre-diagnosis Physical Activity and All-Cause/Colorectal Cancer/Cardiovascular Mortality in Colon and Rectal Cancer Patients.

Treatment	PA	*n*	Person-years	All-Cause			Colorectal Cancer			Cardiovascular		
				Events	HR	(95% CI)	Events	HR	(95% CI)	Events	HR	(95% CI)
**Colon cancer**												
		27,143	66,458.54	1673			1155			51		
Total	low[a]	12,755	31,036.73	791	1 (Ref)		548	1 (Ref)		29	1 (Ref)	
	high[b]	14,388	35,421.80	882	0.86	(0.77, 0.95)	607	0.87	(0.77, 0.98)	22	0.49	(0.27, 0.87)
		20,231	51,613.34	754			380			44		
Surgery only	low	9546	24,127.51	364	1 (Ref)		188	1 (Ref)		23	1 (Ref)	
	high	10,685	27,485.83	390	0.77	(0.66, 0.90)	192	0.75	(0.61, 0.93)	21	0.60	(0.32, 1.12)
		6721	14,495.30	841			703			7		
Surgery with chemotherapy and/or radiotherapy	low	3114	6747.56	392	1 (Ref)		328	1 (Ref)		6	1 (Ref)	
	high	3607	7747.74	449	0.94	(0.82, 1.09)	375	0.96	(0.82, 1.12)	1	0.09	(0.01, 0.78)
		191	349.90	78			72			0		
Chemotherapy with or without radiotherapy	low	95	161.67	35	1 (Ref)		32	1 (Ref)		0		
	high	96	188.23	43	0.93	(0.57, 1.52)	40	0.88	(0.53, 1.48)	0		
**Rectal cancer**												
		16,453	40,468.54	971			686			27		
Total	low	8049	19,735.77	495	1 (Ref)		347	1 (Ref)		11	1 (Ref)	
	high	8404	20,732.77	476	0.86	(0.75, 0.98)	339	0.92	(0.79, 1.07)	16	1.17	(0.53, 2.57)
		9993	26,008.18	318			148			19		
Surgery only	low	4828	12,547.87	156	1 (Ref)		70	1 (Ref)		7	1 (Ref)	
	high	5165	13,460.31	162	0.82	(0.65, 1.03)	78	0.97	(0.69, 1.35)	12	1.30	(0.50, 3.40)
		5966	13,666.00	552			447			8		
Surgery with chemotherapy and/or radiotherapy	low	2992	6824.21	287	1 (Ref)		229	1 (Ref)		4	1 (Ref)	
	high	2974	6841.79	265	0.87	(0.73, 1.04)	218	0.92	(0.76, 1.12)	4	0.84	(0.20, 3.57)
		494	794.36	101			91			0		
Chemotherapy with or without radiotherapy	low	229	363.70	52	1 (Ref)		48	1 (Ref)		0		
	high	265	430.67	49	0.99	(0.64, 1.51)	43	0.92	(0.59, 1.44)	0		

Analysis with pre-diagnosis physical activity. [a] Patients with a low level of activity (the weighted sum of the frequencies for walking, moderate, and vigorous activity less than 3 times/week). [b] Patients with a high level of activity (the weighted sum of the frequencies for walking, moderate, and vigorous activity greater than or equal to 3 times/week).

Table S3 Change of Physical Activity Levels before and after diagnosis and All-Cause/Colorectal Cancer/Cardiovascular Mortality in Colon and Rectal Cancer Patients.

Treatment	PA	*n*	Person-years	All-Cause			Colorectal Cancer			Cardiovascular		
				Events	HR	(95% CI)	Events	HR	(95% CI)	Events	HR	(95% CI)
**Colon cancer**												
Total		27,143	66,458.54	1673			1155			51		
	constant	17,065	41,857.62	984	1 (Ref)		669	1 (Ref)		33	1 (Ref)	
	decreased	4159	10,172.22	316	1.29	(1.14, 1.47)	213	1.30	(1.11, 1.52)	6	0.66	(0.28, 1.59)
	increased	5919	14,428.70	373	1.18	(1.04, 1.33)	273	1.23	(1.07, 1.42)	12	1.26	(0.65, 2.45)
Surgery only		20,231	51,613.34	754			380			44		
	constant	12,734	32,609.12	452	1 (Ref)		228	1 (Ref)		29	1 (Ref)	
	decreased	3142	8012.62	145	1.22	(1.01, 1.47)	68	1.14	(0.87, 1.50)	6	0.74	(0.31, 1.79)
	increased	4355	10,991.59	157	1.17	(0.98, 1.40)	84	1.21	(0.94, 1.56)	9	1.09	(0.52, 2.32)
Surgery with chemotherapy and/or radiotherapy		6721	14,495.30	841			703			7		
	constant	4220	9048.68	484	1 (Ref)		397	1 (Ref)		4	1 (Ref)	
	decreased	974	2076.94	153	1.38	(1.15, 1.66)	128	1.42	(1.16, 1.73)	0		
	increased	1527	3369.67	204	1.18	(1.00, 1.40)	178	1.24	(1.04, 1.48)	3	2.52	(0.55, 11.51)
Chemotherapy with or without radiotherapy		191	349.90	78			72			0		
	constant	111	199.81	48	1 (Ref)		44	1 (Ref)		0		
	decreased	43	82.67	18	0.98	(0.55, 1.74)	17	1.01	(0.56, 1.83)	0		
	increased	37	67.43	12	0.74	(0.38, 1.44)	11	0.76	(0.38, 1.52)	0		
**Rectal cancer**												
Total		16,453	40,468.54	971			686			27		
	constant	10,145	25,021.27	569	1 (Ref)		389	1 (Ref)		21	1 (Ref)	
	decreased	2578	6220.89	185	1.22	(1.03, 1.44)	139	1.32	(1.09, 1.61)	3	0.52	(0.16, 1.77)
	increased	3730	9226.38	217	1.05	(0.90, 1.23)	158	1.09	(0.91, 1.32)	3	0.40	(0.12, 1.33)
Surgery only		9993	26,008.18	318			148			19		
	constant	6241	16,237.38	183	1 (Ref)		78	1 (Ref)		15	1 (Ref)	
	decreased	1508	3894.49	64	1.26	(0.94, 1.68)	37	1.73	(1.16, 2.57)	2	0.49	(0.11, 2.19)
	increased	2244	5876.30	71	1.15	(0.87, 1.51)	33	1.26	(0.84, 1.89)	2	0.38	(0.09, 1.68)
Surgery with chemotherapy and/or radiotherapy		5966	13,666.00	552			447			8		
	constant	3616	8299.93	329	1 (Ref)		259	1 (Ref)		6	1 (Ref)	
	decreased	972	2193.53	99	1.13	(0.90, 1.42)	83	1.20	(0.94, 1.54)	1	0.58	(0.07, 4.84)
	increased	1378	3172.54	124	1.04	(0.84, 1.28)	105	1.10	(0.87, 1.38)	1	0.50	(0.06, 4.16)
Chemotherapy with or without radiotherapy		494	794.36	101			91			0		
	constant	288	483.96	57	1 (Ref)		52	1 (Ref)		0		
	decreased	98	132.87	22	1.18	(0.70, 2.00)	19	1.10	(0.63, 1.94)	0		
	Increased	108	177.53	22	0.87	(0.52, 1.45)	20	0.86	(0.50, 1.47)	0		

Analysis with change of physical activity level.
